# Autophagy responsive intra-intercellular delivery nanoparticles for effective deep solid tumor penetration

**DOI:** 10.1186/s12951-022-01514-6

**Published:** 2022-06-25

**Authors:** Fengling Wang, Dandan Xie, Wenjing Lai, Min Zhou, Jie Wang, Rufu Xu, Jingbing Huang, Rong Zhang, Guobing Li

**Affiliations:** Present Address: Department of Pharmacy, The Second Affiliated Hospital of Army Medical University, No. 183 Xinqiao Road, Chongqing, China

**Keywords:** Autophagy responsive, Transcellular transport, Deep penetration, Solid tumors

## Abstract

Deep tumor cells (cells in the center of solid tumors) play a crucial role in drug tolerance, metastasis, recurrence and microenvironment immune suppression. However, their deep location endows them with an untouched abdomen and makes them refractory to current treatments. Herein, we exploited the characteristic of higher autophagy in deep tumor cells than in superficial tumor cells and designed autophagy-responsive multifunctional nanoparticles (PGN) to enhance drug accumulation in deep tumor cells. PGNs were prepared by densely coating poly (lactic-co-glycolic acid) (PLGA) with cationic autophagy-responsive cell-penetrating peptide (GR9) and anionic 2,3-dimethylmaleic anhydride (DMA)-modified DSPE-PEG. The suitable nanoparticle size (122.4 nm) and charge-neutral surface (0.21 mV) of the NPs enabled long blood circulation. The hydrolysis of surface-anchored anionic DMA in the acidic microenvironment led to the exposure of the GR9 peptide and enhance tumor penetration. Once the PGN arrived in deep tumor cells with strong autophagy, GR9 was cut off by an autophagy shear enzyme, and the nanoparticles remained in the cells to undergo degradation. Furthermore, we prepared docetaxel (DTX) and chloroquine (CQ) loaded d-PGN. CQ inhibits autophagosome fusion with lysosomes, resulting in autophagosome accumulation, which further enhances the sensitivity of d-PGN to autophagy and their deep tumor retention. In vivo experiments showed that drug-loaded d-PGN achieved excellent antitumor efficacy with a peak inhibition rate of 82.1%. In conclusion, autophagy-responsive multifunctional nanoparticles provide a novel potential strategy for solid tumor treatment.

**Supplementary Information:**

The online version contains supplementary material available at 10.1186/s12951-022-01514-6.

## Introduction

Deep tumor cells (cells in the center of solid tumors) play a crucial role in drug tolerance, metastasis and microenvironment immune suppression [1, 2]. However, the deep location of deep tumor cells leaves them unaffected by treatments. Recently, the deep tumor cell-oriented delivery of nanodrugs has emerged as a promising strategy to improve antitumor efficiency [3]. However, multiple physiological and biological barriers present great challenges to targeting deep tumor cells [4, 5].

Traditional strategies to improve tumor penetration mainly include modulating the tumor microenvironment or optimizing nanoparticle properties, but these traditional strategies still have limitations. The modulation of tumor microenvironments by external forces might disrupt the balance of the tumor microenvironment and cause tumor metastasis [6]. Optimizing nanoparticle properties, such as constructing shrinkable nanoparticles, equipping specific ligands or monoclonal antibodies or carbohydrates, designing reversible charge nanoparticles, and so on, have shown promising prospects for tumor treatment [7–12]. However, it is challenging to integrate all the desired specialized functions into a single drug delivery nanoparticle, and the above strategies are still inefficient for targeting deep tumors. Therefore, more advanced strategies could be explored to overcome these limitations. Triarylphosphine (TPP)-mediated transcellular transport could overcome the obstruction of the tumor stroma and might be a promising approach to enhance tumor penetration [13]. For example, the classic transmembrane peptide R9 can directly enter cells by electrostatic action [8]. Moreover, R9 contains several guanidine structures and can thus achieve lysosome escape, making it a good candidate to enhance tumor penetration [14]. However, its therapeutic effectiveness is impeded by a lack of specificity, which prevents a concentrated distribution in the tumor area. Therefore, it might greatly improve the tumor therapeutic effect to endow R9 with the ability to target transcellular transport in deep tumor cells.

It has been reported that deep tumor cells have a higher level of autophagy than superficial tumor cells and rely on autophagy to resist the pressure from a lack of nutrition, radiotherapy and chemotherapy drugs and thus maintain their steady state [15–17]. Moreover, autophagy is a relatively independent system in cells with a series of unique enzymes, such as ATG4B, one of the most important autophagy-specific enzymes, which is highly expressed in deep tumor cells [17, 18]. The GTFGF sequence is the peptide fragment of the LC3 protein, which is the specific substrate of the autophagy enzyme ATG4B and can be cleaved by ATG4B [19]. Wang Hao’s research group used the GTFGF sequence to connect fluorescent molecules to nanoparticles and monitor the autophagy level, as the nanoparticles could generate fluorescence signal changes in response to the autophagy level by cleavage, proving that the GTFGF sequence had a strong sensitivity to autophagy enzymes [20, 21]. Based on these considerations, we propose conjugating the GTFGF sequence to R9 to construct the multifunctional tandem peptide GR9. Using GR9-modified nanoparticles may endow the nanoparticle with autophagy-responsive transmembrane penetration ability to achieve deep tumor-targeted delivery. To our knowledge, accurately controlling drug delivery in deep tumor cells by changing the transport pathway of a nanodrug delivery system through autophagy has not yet been reported. However, the positive charge of GR9 might lead to rapid elimination by the immune system, thereby restricting it’s in vivo application [22]. Anionic 2,3-dimethylmaleic anhydride (DMA) is widely reported as a good candidate to shield the positive charge. Moreover, the pH-responsive hydrolysis ability of DMA could expose GR9 at an appropriate point to increase deep penetration upon reaching the tumor tissue. Therefore, using DMA to shield the positive charge of GR9 would be favorable for tumor-targeting nanoparticles [23, 24].

**Fig. 1:**
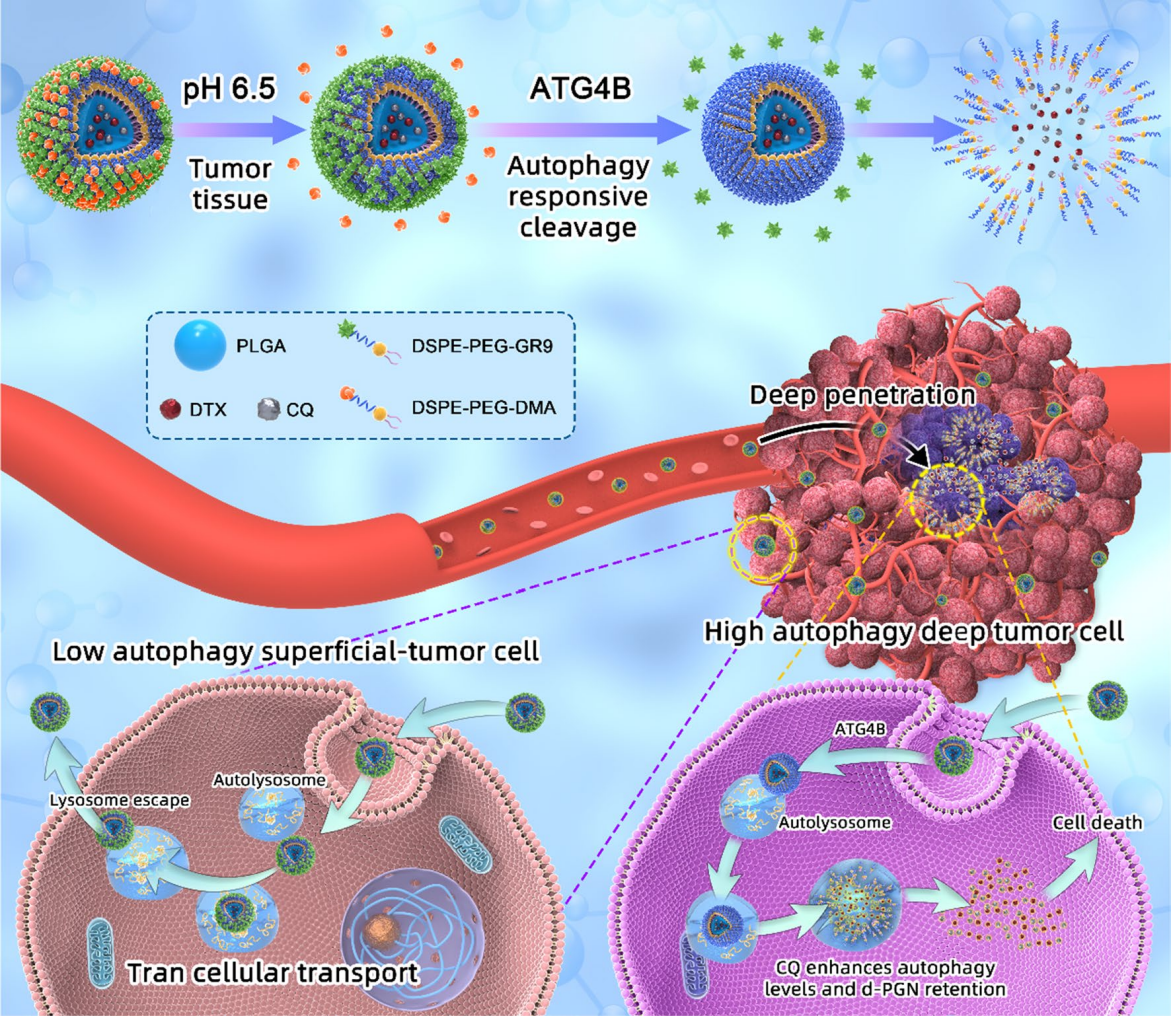
Schematic diagram. Schematic illustration of the self-assembly and transportation pathway of autophagy responsive intra-intercellular delivery nanoparticles

In this study, we reported autophagy-responsive multifunctional nanoparticles (PGN), which could deliver drugs to deep tumor cells by targeted transcellular transport (Fig. 1, schematic diagram). In detail, PGN is prepared by densely coating poly (lactic-co-glycolic acid) (PLGA) with a cationic autophagy-responsive cell-penetrating peptide (GR9) and anionic 2,3-dimethylmaleic anhydride (DMA)-modified DSPE-PEG. The drug docetaxel (DTX) and the autophagy degradation inhibitor chloroquine (CQ) were further loaded. We hypothesized that the nanoparticles were nearly electrically neutral in the blood circulation and could expose the positively charged membrane-penetrating peptide to achieve tumor penetration immediately upon arriving in the mildly acidic tumor tissues (pH 6.5). Subsequently, in deep tumor cells with strong autophagy, the membrane penetrating peptide was cut off by autophagy shear enzyme, and the nanoparticles remained in the cells to degrade and release drugs. Then, the released drug CQ could inhibit autophagosome fusion with lysosomes, resulting in autophagosome accumulation, further enhancing the autophagy-responsive sensitivity and deep tumor retention of PGN. By combining the drug CQ and autophagy-responsive nanoparticles with high sensitivity, we hoped to enable PGN to distinguish autophagy conditions and to accumulate in deep tumor cells with high levels of autophagy, thus exerting an outstanding antitumor effect.

## Materials and methods

### Materials

MDSPE-PEG2000, DSPE-PEG2000-NH_2_, and DSPE-PEG2000-NHS were purchased from Peng Sheng Biological (Shanghai, China). The GR9 peptide and R9 peptide were chemically synthesized by GL Biochem (Shanghai, China). Poly (lactic-co-glycolic acid) (PLGA, 12 kDa) was purchased from LACTEL Absorbable Polymers. Coumarin 6 (C_6_), CQ and DTX were all purchased from Sigma-Aldrich. ATG4B was purchased from Biolead (Beijing, China). 3-Methyladenine (3-MA) and rapamycin were purchased from MCE. 4,6-Diamidino-2-phenylindole (DAPI) and lysosome red fluorescence probes were purchased from Beyotime Biotechnology (Shanghai, China).

### Synthesis of DSPE-PEG2000-GR9, DSPE-PEG2000-R9 and DSPE-PEG2000-DMA

Fifty milligrams of DSPE-PEG2000-NHS and 35.8 mg of GR9 or R9 peptide were dissolved in 5 mL of dimethyl sulfoxide (DMSO), and added to 5 µL of triethylamine, and then stirred at moderate speed for 24 h at room temperature [25, 26]. The resulting products were purified by dialysis, lyophilized, and stored at − 20 °C. DSPE-PEG2000-DMA was synthesized by conjugating amino groups of DSPE-PEG2000-NH_2_ to 2,3-dimethylmaleic anhydride (DMA) in phosphate-buffered saline (PBS, pH 8.5) under stirring at moderate speed for 24 h at room temperature. The resulting products were purified by dialysis, finally lyophilized and stored at − 20 °C.

### Preparation and characterization of nanoparticles (NPs)

Nanoparticles (NPs) were prepared by the self-assembly nanoprecipitation method. Briefly, to prepare PN, PLGA (3.0 mg), DSPE-PEG (1.5 mg), and soybean phospholipid (1.3 mg) were dissolved in 150 µL of dimethyl sulfoxide (DMSO). Then, the DMSO mixture was slowly added to 3.0 mL of deionized water under stirring (900 rpm) at room temperature. Finally, the residual DMSO was removed by dialysis in deionized water. Similarly, to prepare PGN, the ratio of DSPE-PEG2000-GR9/DSPE-PEG2000-DMA was changed from 3/2 to 1/2 and used to screen charge-neutral NPs. The fluorescence-labeled NPs were prepared by the same method, except that the hydrophobic fluorescent dye C6 or Did (0.15% w/w) was mixed with the DMSO solution before the addition to deionized water. The size and zeta potential of various NPs were estimated by a Malvern Zeta sizer Nano ZS90. The morphology of the NPs was observed via transmission electron microscopy (TEM).

### Stability and autophagy-responsiveness study

To investigate the in vitro stability of NPs, the collected NPs were dispersed in PBS (pH 7.4) and incubated in a shaker at 37 °C. The particle size was measured at different time points as described above, and the enzyme-responsive behavior of the PGN was then verified. GR9 was labeled with FITC, and PLGA was loaded with rhodamine, which formed a FRET pair. The fluorescence emission spectra were measured for 0, 4, or 6 h at an excitation wavelength of 490 nm [27].

### Cell experiments

#### Cell culture

Mouse melanoma B16F10 cells were cultured in DMEM supplemented with 10% FBS and 1% penicillin–streptomycin in a 5% CO_2_/95% air atmosphere at 37 °C.

#### Cellular uptake study

Flow cytometry and microscopy were used to investigate the cellular uptake of NPs on B16F10 cells. B16F10 cells were first seeded into 12-well plates (1 × 10^5^ cells/well). After culturing for 24 h, the cells were incubated with C6-labeled PN, PRN, and PGN. Then, the PRN and PGN were preincubated with the enzyme ATG4B (equivalent C6 concentration: 2.5 µg/mL) for 2 h. After washing with PBS three times, the cells were finally collected and resuspended in cold PBS to measure the fluorescence intensity by a flow cytometer. For qualitative analysis, as in the previous step, after washing the cells, 4% paraformaldehyde was used to fix the cells for 15 min, and then DAPI (5 mg/mL) was used to stain the nuclei for 5 min. Finally, the cell fluorescence was observed by CLSM.

#### Lysosomal escape

To investigate whether GR9- or R9-modified NPs could escape from lysosomes, we used LysoTracker Red to trace the lysosomes. Briefly, B16F10 cells were first seeded into 12-well plates (3 × 10^4^ cells/well). After culturing for 24 h, the cells were incubated with C6-labeled PN, PGN or PRN (equivalent C6 concentration: 2.5 µg/mL) for 4 h. After washing the cells, LysoTracker Red (75 nM) was incubated with the cells for 30 min at 37 °C. The cells were washed with PBS again, fixed with 4% paraformaldehyde for 15 min, and then stained with DAPI (5 mg/mL) for 5 min. Finally, the cell fluorescence was observed by CLSM.

#### Specific uptake of PGN in autophagy-active cells

To verify the specific uptake of PGN by autophagy-active cells, cells were cotreated with nanoparticles and the autophagy inducer rapamycin, and then the cellular uptake of nanoparticles was quantitatively and qualitatively analyzed. B16F10 cells were first seeded into 12-well plates (1 × 10^5^ cells/well), and some cells were treated with rapamycin (50 nM). After culturing for 24 h, the cells were incubated with PN, PRN or PGN as described above for 2 h (equivalent C6 concentration: 2.5 µg/mL). Then, the cells were washed with PBS three times and cultured for 6 h. Finally, the cells were collected and resuspended in PBS to measure the fluorescence intensity by a flow cytometer. Qualitative analysis was performed as described in the previous step.

#### In vitro transcellular transport ability

To verify the transcellular transport ability of PGN, B16F10 cell monolayers were seeded on transwell inserts fitted with polycarbonate membranes, and some cells were treated with rapamycin (50 nM). After the whole polycarbonate membranes were covered with cells, the apical solution was replaced with 200 µL of PN, PRN or PGN (equivalent C6 concentration: 2.5 µg/mL). After incubation for 6 h, the fluorescence intensity of transported NPs in the basolateral chamber was assessed by a Varioskan Flash.

#### Tumor spheroid penetration ability of nanoparticles

A total of 1000 B16F10 cells were seeded into 96-well plates coated with 50 µL of 2% low melting point agarose. After 5 d, the tumor spheroids were treated with PN, PRN or PGN for 4 h at a Did concentration of 1.5 µg/mL. Then, the tumor spheroids were washed with PBS three times and fixed with 4% paraformaldehyde for 30 min. Finally, the tumor spheroid fluorescence was observed by tomoscan under CLSM.

#### Preparation and characterization of drug-loaded NPs

To obtain drug-loaded NPs, PLGA, CQ, DTX, soybean phospholipid and a desired amount of MDSPE-PEG2000, DSPE-PEG2000-GR9/R9, or DSPE-PEG2000-DMA were dissolved in 150 µL of dimethyl sulfoxide (DMSO), and drug-loaded NPs were prepared by the self-assembly nanoprecipitation method. Then, the NPs were collected through centrifugation with an ultrafiltration tube at 5000 rpm for 40 min. Finally, the drug loading content of DTX and CQ was determined by the high-performance liquid chromatography (HPLC) with a Diamonsil C18 column (150 × 4.6 mm^2^, 5 μm). The mobile phase for DTX was acetonitrile-water (55:45), whereas the mobile phase for CQ was acetonitrile-water (2:8) containing 0.1 M KH_2_PO_4_, 5 mM triethylamine and 5 mM heptane sulfonate sodium [28, 29].

#### Hemolysis assay

Whole blood from a healthy SD rat was collected in heparinized tubes, then centrifuged at 5000×*g* for 5 min to get RBCs. The collected RBCs were divided into different groups and added with 1 mL of nanoparticle solutions with 15.6, 31.3, 62.5, 125, 250, and 500 µg/mL concentrations. After incubating at 37 °C for 3 h, tubes were centrifuged to get 100 µL supernatant, and the absorbance was measured at 541 nm. The RBC hemolysis ratio was calculated by the formula below. The experiment was duplicated three times. Hemolysis % = [(OD test − OD negative control)/(OD positive control − OD negative control)] ∈ 100.

#### TEM imaging of autophagosomes in B16F10 cells

B16F10 cells were treated with different formulations for 24 h and collected to obtain cell pellets. Then, the cell pellets were fixed with 2.5% glutaraldehyde for 24 h, and 2% osmium tetroxide was added for another 30 min of fixation. Finally, ultrathin sections of B16F10 cell pellets were observed by JEM-1200EX TEM.

#### CLSM observation of fluorescent LC3 in B16F10 cells

mRFP-EGFP-LC3-expressing B16F10 cells were constructed and imaged by CLSM to analyze autophagic flux after treatment with different formulations.

#### In vitro antitumor activity

The cytotoxicity of free drugs and NPs was investigated in B16F10 cells under 2D culture conditions or in 3D tumor spheroids using the MTT assay. First, B16F10 cells were seeded onto 96-well plates (3D tumor spheroids with 2% low melting point agarose), treated with free drugs or NPs for 24 h, and incubated with MTT (5 mg/mL, 20 µL per well) for another 4 h. Finally, the formazan of MTT was dissolved by dimethyl sulfoxide (150 µL per well), and then the absorbance was measured using Varioskan Flash.

#### Apoptosis

Annexin V-FITC/PI staining was used to evaluate the apoptosis-inducing ability of different free drugs and nanoparticles. Briefly, B16F10 cells were seeded into 12-well plates, incubated for 24 h, and then treated with free drugs or NPs for 24 h. After staining with Annexin V-FITC/PI for 15 min, the fluorescence intensity was analyzed by flow cytometry

#### Microtubule polymerization analysis

Briefly, B16F10 cells were first seeded into 12-well plates (5 × 10^4^ cells/well). After culturing for 24 h, the cells were incubated with free drugs or NPs for 24 h, then fixed with 4% paraformaldehyde and rinsed with 1% bovine serum albumin (BSA) in phosphate-buffered saline (PBS), then incubated with antibody against β-tubulin (1:100) overnight at 4 °C. After washing the cells, cells were incubated with Cy3-conjugated mouse anti-rabbit IgG for 2 h at room temperature, then stained with DAPI (5 mg/mL) for 5 min. Finally, the cell fluorescence was observed by CLSM.

### Animal studies

Female C57BL/6 mice (20 ± 2 g) were obtained from the Laboratory Animal Center of the Third Military Medical University. All animal experiments were approved by the Laboratory Animal Welfare and Ethics Committee of the Third Military Medical University (assigned number: AMUWEC2020477).

#### In vivo imaging

The tumor distribution of PNs was investigated in B16F10 melanoma tumor-bearing C57 mice. The tumor-bearing mice were intravenously administered Did containing PN, PRN, or PGN at a dose of 0.8 mg/kg (Did equally). At 24 h after injection, the mice and the harvested tumors were observed under a near-infrared reflection fluorescence imaging system. Then, tumor tissues were fixed in 10% formaldehyde for several days, embedded in paraffin and sectioned with a microtome. To analyze tumor permeability, tumor vessels were labeled with FITC-conjugated CD31 antibody (green signal), and samples were imaged using confocal microscopy. To analyze the LC3 expression level, the cell nuclei were stained with DAPI, and LC3 was labeled with FITC-conjugated CD31 antibody and analyzed by immunofluorescence.

#### In vivo antitumor efficacy and biosafety

The in vivo antitumor efficacy of NPs or free drug was investigated in B16F10 melanoma tumor-bearing C57 mice. When the tumors reached 100 mm^3^, the mice were randomly divided into seven groups (eight mice per group) and intravenously administered saline, CQ, DTX, CQ/DTX, d-PN, d-PRN or d-PGN (2 mg/kg DTX equivalents) on Days 5, 6, 7 and 8. The tumor size and body weight were measured every day by vernier caliper or electronic balance. The tumor size was calculated by the formula: *Volume* = (*Length* ∈ *Width*^2^)/2. Finally, blood was obtained from the eye socket, and the mice were sacrificed to obtain the tumors. The serum separated from the ocular blood was used to assess the biochemical factors (AST, ALT, CK, LDH, urea and CREA).

#### Histological analysis

For histological analysis, the organs, including tumor, liver, spleen, heart, lung, and kidneys, were fixed in 4% paraformaldehyde and embedded in paraffin blocks. Then, each section was cut into ultrathin slices and evaluated by hematoxylin–eosin (H&E) and terminal deoxynucleotidyl transferase-mediated dUTP nick end labeling (TUNEL) assays. In addition, the LC3 expression level was analyzed by immunofluorescence. The cells were visualized with an optical microscope (inverted microscope DMi8, Leica).

### Statistical analysis

Data are presented as the mean ± standard deviation and were analyzed by Social Sciences (SPSS) software. Statistical analysis was performed using one-way analysis of variance (ANOVA). Significance differences were defined as *P < 0.05 and **P < 0.01.

## Results and discussion

### Preparation and characterization of nanoparticles

#### Synthesis and characterization of DSPE-PEG2000-GR9/R9 and DSPE-PEG2000-DMA

The D-oligoarginine peptide (R9), a classical cell-penetrating peptide (CPP) that has shown evidence of superior efficiency for transcellular transport [30], was conjugated to the GTFGF sequence to develop the multifunctional peptide GR9, which could respond to a high autophagy level by cleavage. First, the GR9 peptide was conjugated to DSPE-PEG2000-NHS to obtain positively charged DSPE-PEG2000-GR9, and the R9 peptide was conjugated to DSPE-PEG2000-NHS to obtain DSPE-PEG2000-R9 as a comparison group. Charge-reversible 2,3-dimethylmaleic anhydride (DMA) was conjugated to DSPE-PEG2000-NH_2_ to obtain negatively charged DSPE-PEG2000-DMA. The ^1^H NMR spectra (Additional file 1: Fig. S1) confirmed the successful formation of DSPE-PEG2000-GR9/R9 and DSPE-PEG2000-DMA, as the proton peaks from the NHS and NH_2_ groups disappeared after the reaction.

#### Preparation and characterization of NPs

The nanoparticles were self-assembled by PLGA, autophagy-responsive cationic membrane penetrating peptide (GR9), and anionic DMA-modified DSPE-PEG through nanoprecipitation. First, we screened different ratios of DSPE-PEG2000-GR9 and DSPE-PEG2000-DMA to obtain neutrally charged PGNs. As shown in Additional file 1: Fig. S2, the ζ potential of PGNs changed from positive to negative with increasing DSPE-PEG2000-DMA, and when the ratio reached 2:3, PGNs had a neutral charge (−0.733 mV). Hence, a ratio of 2:3 (DSPE-PEG2000-GR9/DSPE-PEG2000-DMA) was used for the subsequent studies. Moreover, the same proportions of DSPE-PEG2000-R9/DSPE-PEG2000-DMA and PLGA were prepared as the PRN group, and MPEG2000-DSPE and PLGA were prepared as the PN group.

**Fig. 2:**
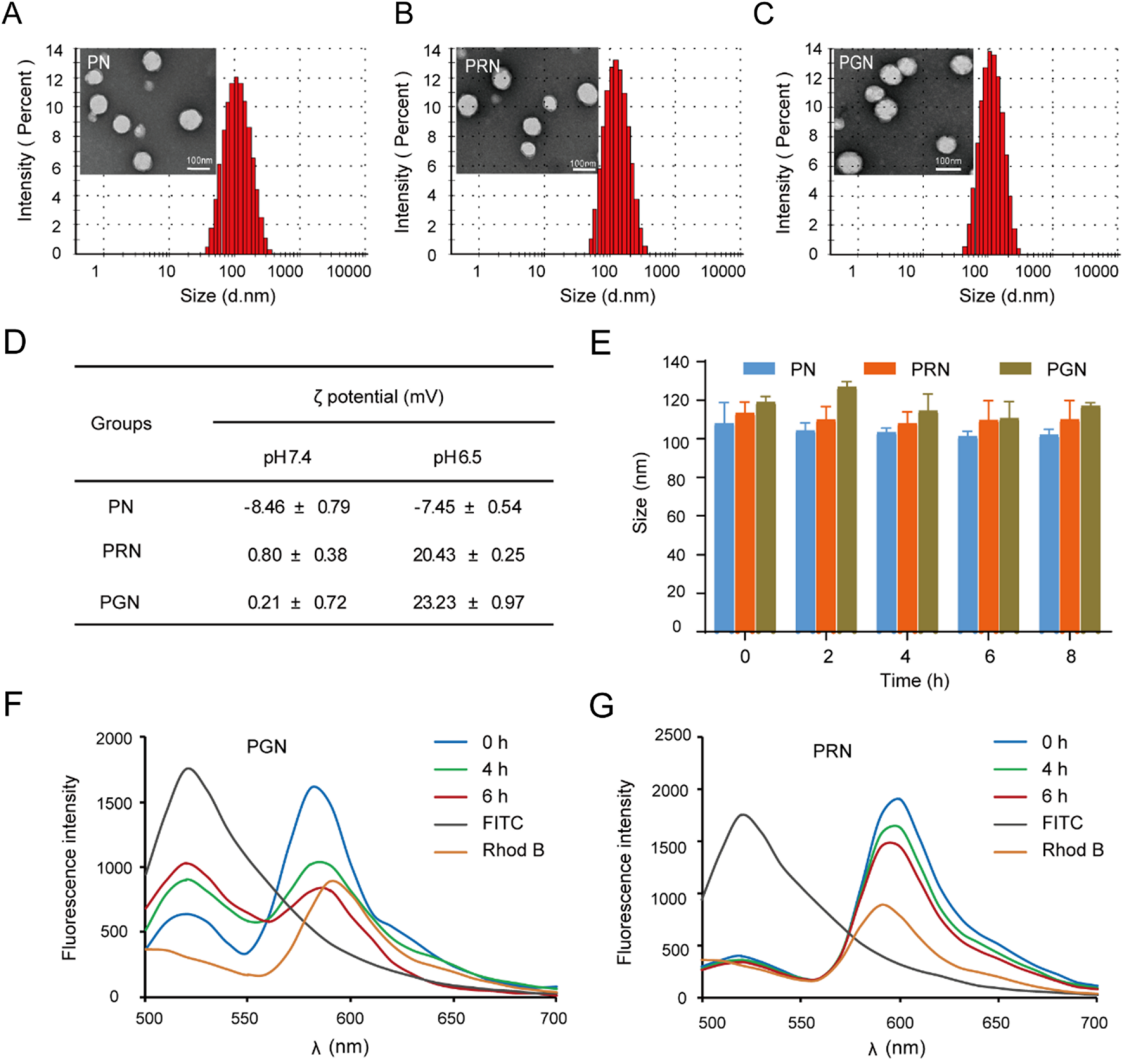
Characterization of NPs. Size distribution and TEM images of **A** PN, **B** PRN and **C** PGN; scale bars: 100 nm. **D** ζ potential change of NPs in phosphate-buffered saline (PBS, pH 7.4 and pH 6.5) for 4 h. **E** Stability of three kinds of NPs in PBS (pH 7.4) at different time points. **F** Fluorescence resonance energy transfer (FRET) spectra of PGN at different times after incubation with ATG4B. Excitation wavelength: 490 nm. **G** Fluorescence resonance energy transfer (FRET) spectra of PRN after different times of incubation with ATG4B. Excitation wavelength: 490 nm.

The hydration and transmission electron microscopy (TEM) images of different NPs showed a well-defined spherical shape and a distinct core-shell structure of each NP with a size of 105.7 nm (polydispersity index (PDI) 0.175). There was a slight increase in size after the co-modification of cationic (GR9/R9) and anionic DMA groups on the surface of the NPs. PRNs and PGN showed sizes of approximately 118.8 nm (PDI 0.162) and 122.4 nm, respectively (Fig. 2A–C). The particle states of the NPs are presented in Additional file 1: Fig. S3, and they all exhibited a similar pale blue opalescence. As we use DMA to shield the positive charge of the cationic peptide, charge reversal occurs when DMA is hydrolyzed in the acidic microenvironment [31]. To test this hypothesis, we evaluated the ζ potential switchability of NPs in a mimetic tumor micro-acidic environment. Consistent with other reports [23, 31], we found a conversion of ζ potential from neutral charge to strong positive charge in PGN and PRN (*P* < 0.05), while there was no obvious change in PN (Fig. 2D). Moreover, the stability experiment results showed that none of the NPs exhibited a significant change in particle size for up to 8 h in PBS solution (*P* > 0.05), indicating their good stability in PBS (Fig. 2E).

#### Autophagy-responsiveness of NPs

To verify the autophagy-responsive behavior of PGN, the spatial interaction between rhodamine-loaded PLGA and FITC-labeled GR9 with or without ATG4B was evaluated by the FRET assay. FITC is a donor, and Rhod B is an acceptor [32], which forms a FRET pair. Theoretically, a strong FRET signal will occur in intact nanoparticles by energy transfer, and the signal will gradually decrease when the nanoparticle decomposes. Herein, PGN or PRN were treated with ATG4B (5 µg/mL), and then the fluorescence emission spectra were measured for 0, 4, or 6 h at an excitation wavelength of 490 nm. As shown in Fig. 2F, the FRET signal of PGN rapidly decreased with increasing incubation time with the ATG4B enzyme, while PRN showed a negligible difference for at least 6 h (Fig. 2G). Therefore, we speculate that GR9 could be cleaved by autophagy enzymes, indicating the superior autophagy-responsiveness of PGN.

### Evaluation of the cellular uptake and distribution of nanoparticles

**Fig. 3:**
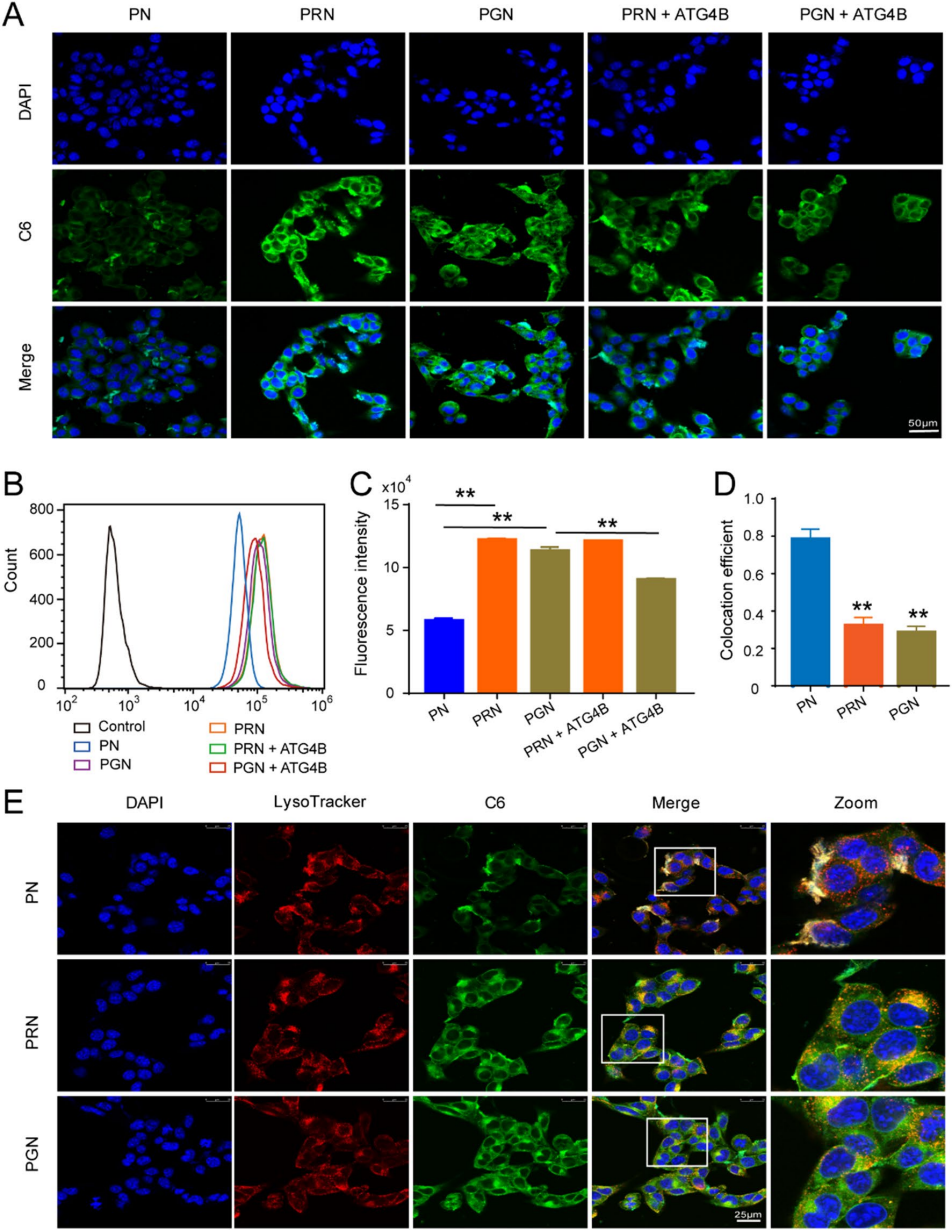
Cellular uptake and distribution of nanoparticles in B16F10 cells. **A** Cellular uptake of NPs after incubation with B16F10 cells for 2 h, as observed by fluorescence microscopy; scale bars: 50 μm. **B**, **C** MFI of the B16F10 cells after treatment with nanoparticles for 2 h. **D** Colocation efficiency of NPs and lysosomes; scale bars: 25 μm. **E** Intracellular lysosome colocalization of NPs in B16F10 cells. Data are presented as the mean ± SD (n = 3). **P < 0.01.

R9 and GR9 can adhere to the cell surface through electrostatic attraction due to their strong positive charge, and the function of promoting cell penetration further enhances cellular uptake [14]. Consistent with these reports, our cellular uptake assay found that the green fluorescence of PRN and PGN was tremendously increased in B16F10 cells compared with that of PN (Fig. 3A). To further investigate the autophagy-responsive behavior of PGN, we also examined the cellular uptake of PGN and PRN under incubation with the autophagy enzyme ATG4B. PGN showed an autophagy-responsive ability, as the cellular uptake of PGN was decreased after incubation with ATG4B, while there was no distinct difference in PRN incubation with ATG4B (Fig. 3A). Similarly, flow cytometry fluorescence results also showed that the cellular uptake of PRN and PGN was 2.13-fold and 2.0-fold higher than that of PN, respectively, whereas ATG4B treatment reduced the fluorescence of intracellular PGN but had no effect on the fluorescence of PRN (Fig. 3B, C). These results confirm that our modification of R9 and GR9 could improve the cellular uptake of NPs and that GR9 confers the autophagy-responsive degradation ability of PGN.

After entering the cells, the NPs are commonly trapped in lysosomes, which are degraded in harsh acidic environments [33]. Therefore, escape from the endosomal/lysosomal region is essential for the therapeutic effects of NPs. Cell-penetrating peptides (CPPs) were reported to promote endosomal/lysosomal escape through the “proton sponge effect” [34]. To verify the lysosomal escape ability of GR9 and R9, lysosomes were labeled with LysoTracker Red, and the lysosome trafficking of NPs in B16F10 cells was observed by confocal fluorescence laser scanning microscopy (CLSM). The results showed that most of the PN were aggregated in lysosomes/endosomes after incubation for 4 h, as evidenced by the red fluorescence of LysoTracker colocalized with the green fluorescence of NPs (Fig. 3D), and exhibited a high colocalization efficiency (Rr) of 0.783 (Fig. 3E). However, more green fluorescence signals of PGN and PRN were observed in the cytoplasm, and the colocalization efficiency significantly decreased (Rr = 0.283 and 0.32), illustrating excellent lysosomal escape ability mediated by GR9 and R9.

### In vitro transcellular transportation and penetration ability of nanoparticles

**Fig. 4:**
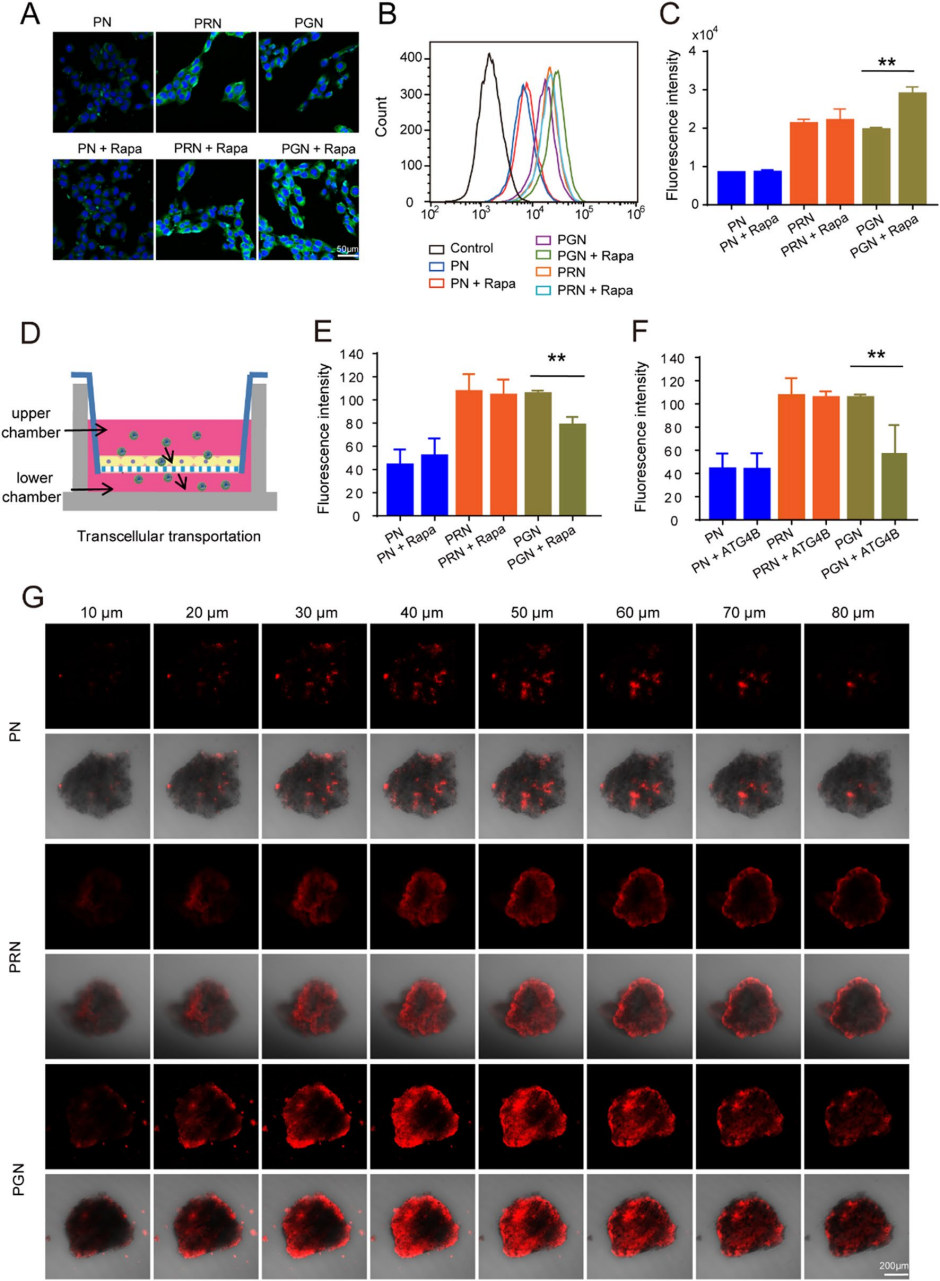
In vitro transcellular transportation and penetration ability of nanoparticles. **A** Qualitative analysis of the fluorescence intensity of NPs staying in cells by fluorescence microscopy; scale bars: 50 μm. **B**, **C** Quantitative analysis of the fluorescence intensity of NPs remaining in the cells by flow cytometry. **D** Schematic illustration of the transcellular transportation of NPs. **E**, **F** Qualitative analysis of the transmembrane ability of nanoparticles. **G** Representative confocal microscopy images of the penetration of PN, PRN and PGN into B16F10 tumor spheroids to different depths. Scale bar: 200 μm. Data are presented as the mean ± SD (n = 3). **P < 0.01.

After NPs entered the cells, the medium containing NPs was removed, followed by incubation with fresh medium for another 6 h. A microscope showed that the intracellular fluorescence intensity of PGN and PRN was much higher than that of the PN (Fig. 4A–C). Interestingly, coculture with the autophagy activator rapamycin significantly enhanced the intracellular fluorescence of PGN but had no effects on PN and PRN (Fig. 4A–C). This was because GR9 could be cleaved immediately in response to autophagy, thus leading to more NPs being retained in highly autophagic tumor cells. Moreover, the transcellular transportation of NPs was evaluated by transwell-permeable supports. As shown in Fig. 4D, after incubation for 6 h, the fluorescence intensity of transported NPs in the basolateral chamber was assessed. Compared with the PN group, PGN and PRN showed superior transcellular transportation ability, which was 2.42-fold (*P* < 0.05) and 2.46-fold (*P* < 0.01) higher than that of PNs (Fig. 4E). These results confirmed that the modification of GR9 and R9 could facilitate the transport of PGN and PRN through the cell monolayer. Moreover, when cocultured with the autophagy activator rapamycin and ATG4B, only the PGN group showed a decreased transportation ability (Fig. 4E, F), further proving that autophagy-responsive PGN nanoparticles could be retained more in autophagy-activated tumor cells.

The B16F10 3D tumor spheroid model includes intercellular interactions and extracellular matrix structures, which can closely mimic in vivo tumors and was employed in our study to predict tumor penetration ability [35]. Consistent with our above results, PN were mostly distributed on the surface of the tumor spheroids. Dramatically deeper spheroid penetration was observed in the PRN and PGN treated groups, as R9 and GR9 could effectively promote the penetration of tumor cells by the NPs (Fig. 4G). Notably, PGN showed higher fluorescence in the center of the tumor than PRNs, which also proved that GR9 has better deep tumor accumulation than R9 because GR9 could be cleaved immediately in response to autophagy-active deep tumor cells, thus leading to the retention of NPs in the center of the tumor. These results suggest that the modification of GR9 endows PGN with high cellular uptake and autophagy-responsive capability, thereby leading more PGN to deeply penetrate the 3D tumor spheroid and exhibiting outstanding tumor cytotoxicity.

### Characterization of drug-loaded NPs

Autophagy is often a protective mechanism to resist external pressure [36]. It has been recognized that blocking the process of autophagy can enhance the anticancer effects of chemotherapeutics, and the combination of autophagy inhibitors and chemotherapeutic drugs has been proposed as a novel therapeutic strategy. Chloroquine (CQ) and its derivative hydroxychloroquine are FDA-approved autophagy degradation inhibitors for cancer patient treatment in the clinic [37, 38]. Therefore, we loaded CQ and the chemotherapy drug docetaxel (DTX) into our prepared NPs and obtained d-PN, d-PRN, and d-PGN, hoping to improve the deep tumor accumulation and antitumor efficacy of the NPs through their synergistic effects. There were slight changes in size and ζ potential after drug loading (Additional file 1: Table S1), and the contents of DTX and CQ in the NPs were determined by HPLC. When the weight of DTX and CQ was 2.5 mg:2.5 mg, there was an optimal encapsulation rate and drug loading. The actual loading ratios of DTX and CQ were controlled at 4.24% and 4.79%, respectively, and they were used in the subsequent experiments.

### Hemocompatibility assay

A hemolysis assay of d-PN, d-PRN and d-PGN was performed to verify the compatibility of NPs with RBCs. As shown in Additional file 1: Fig. S4A, none of the NPs caused hemolysis at the experimental concentration. The amount of hemoglobin released from RBCs into the solution was quantified to evaluate the degree of hemolysis, and the hemolysis rate of all NPs was less than 2% (Additional file 1: Fig. S4B), indicating that d-PN, d-PRN and d-PGN possess excellent hemocompatibility.

### Drug-loaded PGNs enhanced intracellular autophagy

**Fig. 5:**
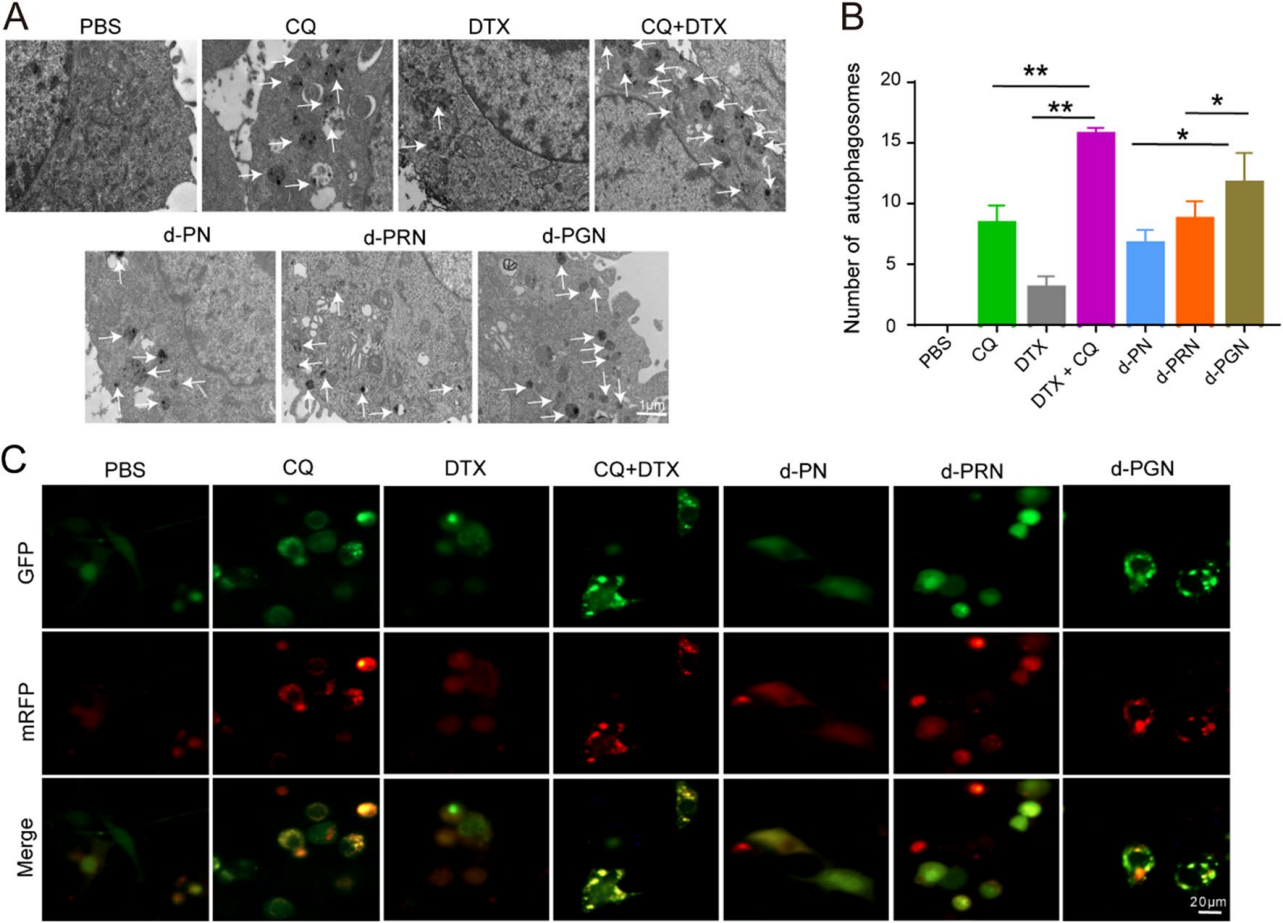
The intracellular autophagy level of NPs in B16F10 cells. **A** TEM images of autophagosomes in B16F10 cells after treatment with free drug and drug-loaded NPs for 24 h (equivalent DTX/CQ concentration: 20 µg/mL), scale bars: 1 μm. **B** Number of autophagosomes in B16F10 cells after treatment with free drug and NPs. **C** CLSM images of autophagosomes in B16F10 cells after treatment with free drug and NPs for 24 h; scale bars: 20 μm.

To verify whether CQ could enhance the intracellular autophagy level, the accumulation of autophagosomes was evaluated. The TEM images revealed that, similar to the combined CQ and DTX treatment group, all drug-loaded NPs markedly increased the accumulation of autophagosomes, especially the d-PGN group (Fig. 5A, B). Autophagic flux in mRFP-GFP-LC3-expressing B16F10 cells after drug treatment was monitored by CLSM. GFP fluorescence exists in early autophagosomes but quenches in late autolysosomes due to the acidic environment, while mRFP is acid-stable so that red fluorescence appears in both early autophagosomes and late autolysosomes [32]. Thus, the red fluorescent puncta represent autolysosomes, and the yellow fluorescent puncta represent autophagosomes. Coincident with the TEM results, CQ treatment greatly increased the yellow puncta compared with the PBS and DTX groups, indicating the accumulation of autophagosomes. Similar results were noted in drug-loaded NPs treated cells, and d-PGN treated cells exhibited many more yellow puncta than d-PRN and d-PN treated cells (Fig. 5C). These results suggest that CQ can increase the PGN-mediated intracellular autophagy level, thereby promoting the cellular retention of d-PGN.

### In vitro therapeutic efficacy of drug-loaded PGNs in B16F10 cells

**Fig. 6:**
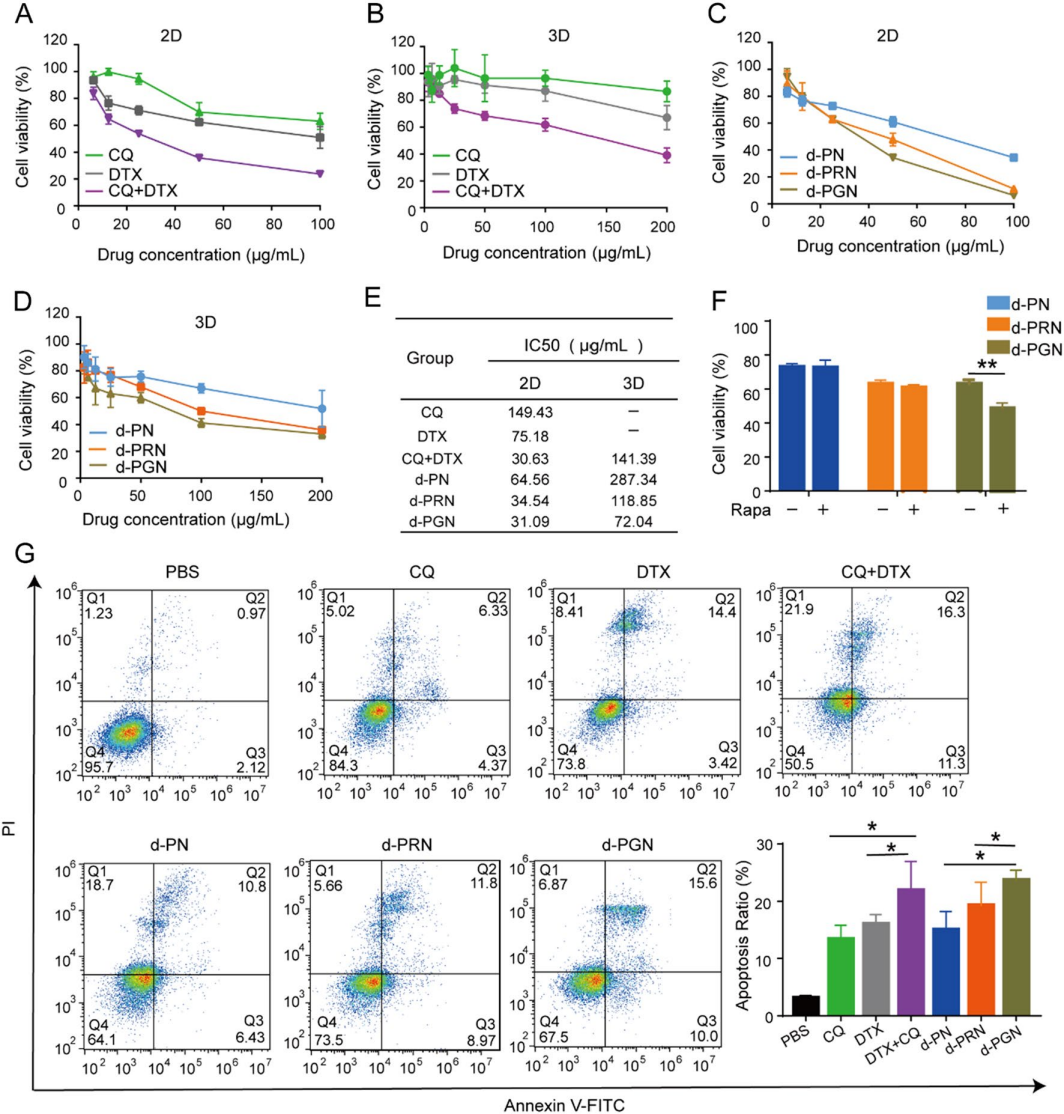
In vitro therapeutic efficacy of d-NPs in B16F10 cells. **A**, **B** The cytotoxicity of the free drug to B16F10 cells in traditional two-dimensional (2D) cell culture (DTX dose of treatment in 2D was 100 µg/mL, 50 µg/mL, 25 µg/mL, 12.5 µg/mL, 6.25 µg/mL) and three-dimensional (3D) tumor spheroids (DTX dose of treatment in 3D was 200 µg/mL, 100 µg/mL, 50 µg/mL, 25 µg/mL, 12.5 µg/mL, 6.25 µg/mL). **C**, **D** The cytotoxicity of nanoformulations (d-PN, d-PRN and d-PGN) to B16F10 cells in traditional 2D cell culture and 3D tumor spheroids. **E** IC_50_ of free drugs and drug-loaded NPs on traditional two-dimensional (2D) cells and 3D tumor spheroids. **F** Cytotoxicity of B16F10 cells cotreated with rapamycin and drug-loaded NPs for 24 h. **G** Apoptosis of B16F10 cells after treatment with free drug and drug-loaded NPs for 24 h.

Next, MTT assays of traditional two-dimensional (2D) cell culture and three-dimensional (3D) tumor spheroids were employed to investigate the cytotoxicity of free drugs and drug-loaded NPs to B16F10 cells. The results showed that both CQ + DTX combined treatment and single-drug treatment caused a dose-dependent decrease in cell viability (Fig. 6A, B). The different IC_50_ values of each group are shown in Fig. 5E. In 2D cell culture, CQ + DTX combined treatment markedly decreased the IC_50_ to 30.63 µg/mL compared with that of CQ (IC_50_: 149.43 µg/mL) or DTX (IC_50_: 75.18 µg/mL) treatment alone, demonstrating that there is a synergistic cytotoxicity to combined treatment with CQ and DTX. After loading CQ and DTX into NPs, we found that the d-PGN group exhibited the strongest cytotoxicity compared with the d-PRN and d-PN groups in both 2D cell culture and 3D tumor spheroids, as evidenced by the IC_50_ values of d-PGN being 31.09 µg/mL (2D) and 72.04 µg/mL (3D), which were much lower than those of d-PRN (2D: 34.54 µg/mL, 3D: 118.85 µg/mL) and d-PN (2D: 64.56 µg/mL, 3D: 287.34 µg/mL) (Fig. 6C–E). To further confirm that the autophagy-responsive ability is still present on drug-loaded PGN, we cotreated B16F10 cells with rapamycin (an autophagy inducer) and drug-loaded NPs. Rapamycin treatment significantly decreased the viability of d-PGN treated B16F10 cells; in contrast, combined treatment with rapamycin and d-PN or d-PRN did not show any significant changes in cell viability (Fig. 6F), suggesting that the superior cytotoxicity of d-PGN was partly due to its autophagy-responsive ability. Furthermore, Annexin V/PI staining was used to investigate the cellular apoptosis induced by the free drug and drug-loaded NPs. Consistent with other reports, CQ causes apoptosis, as CQ can increase the accumulation of autophagosomes by inhibiting autophagic degradation [39, 40]. Similar results were observed in which combined treatment with CQ and DTX significantly increased cell apoptosis compared with DTX or CQ treatment alone. More importantly, the synergistic cytotoxicity of the combined administration of CQ and DTX was also observed in drug-loaded NP, and d-PN, d-PRN, and d-PGN induced 15.04%, 19.29%, and 23.65% cell apoptosis, respectively. As expected, d-PGN caused the highest proportion of cell apoptosis compared to the other groups (Fig. 6G). It was reported that DTX exerts its anti-tumor therapeutic effect by inhibiting microtubule polymerization. We then examined the morphology of microtubule after d-PN, d-PRN, and d-PGN treatment. The results showed that three drug-loaded NPs and free DTX treatment caused severely abnormal microtubule polymerization (Additional file 1: Fig. S5). Taken together, these findings suggest that our nanoparticle encapsulation does not affect the efficacy of DTX and CQ, and effectively exploits their synergistic effects.

### Tumor accumulation and distribution of did-labeled PGN in B16F10 tumor-bearing mice

**Fig. 7:**
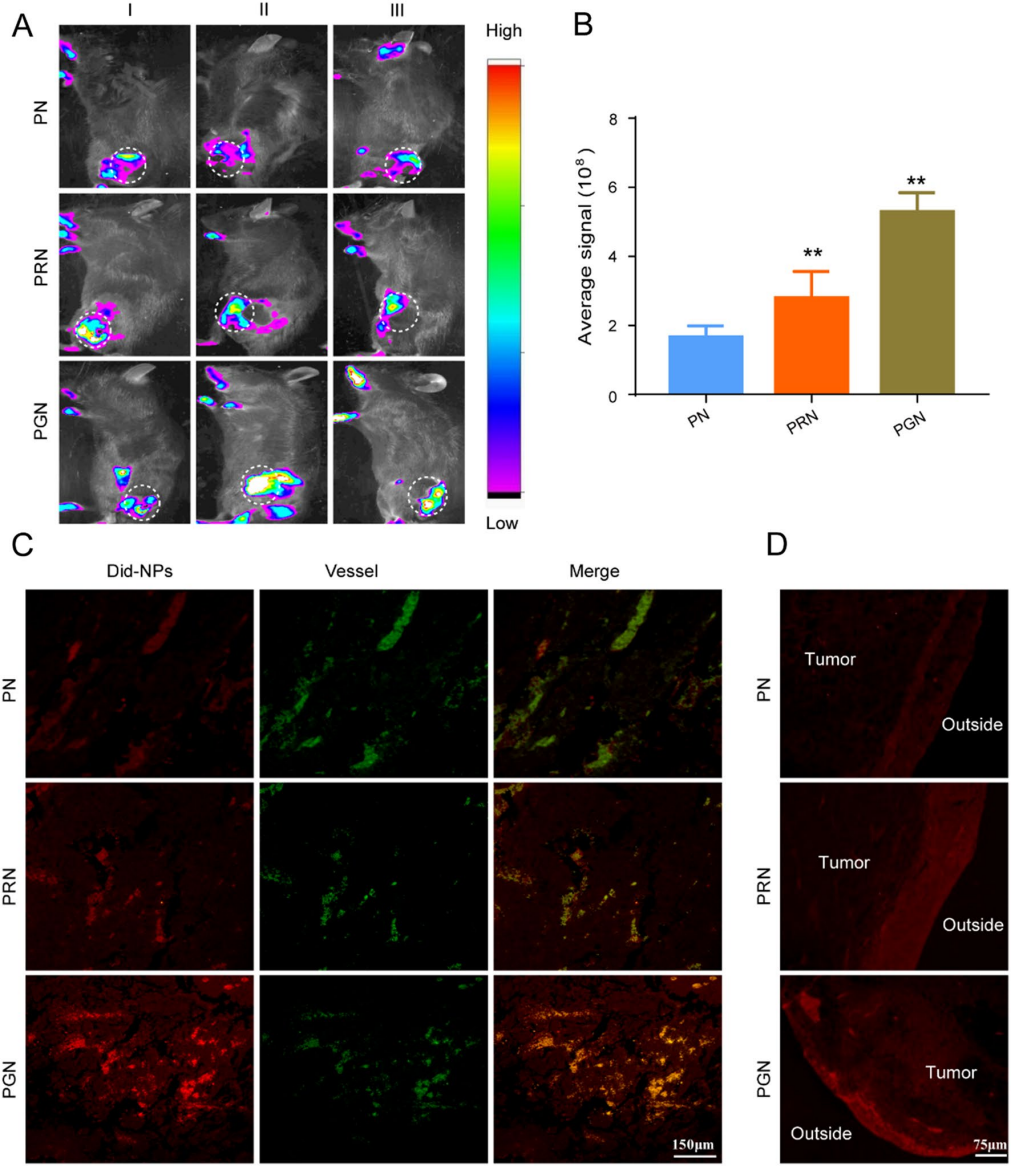
Tumor accumulation and distribution of PGN in B16F10 tumor-bearing mice. **A** Accumulation of PN, PRN and PGN in tumor tissues. **B** Ex vivo tumor tissue images. **P < 0.01 vs. d-PN. **C** The distribution of PN, PRN and PGN in tumor tissues and tumor vessels labeled with FITC-conjugated CD31 antibody (green signal); scale bars: 150 μm. **D** The distribution of drug-loaded NPs (red signal) on the edge and inside area of tumor sections; scale bars: 75 μm.

To verify whether GR9-mediated deep tumor penetration could be replicated in vivo, the accumulation and distribution of Did-labeled NPs in B16F10 tumor-bearing mice were evaluated by in vivo imaging experiments. PRN and PGN showed higher fluorescence signals in the tumor site than PNs, indicating that the modification of GR9 and R9 effectively caused deep tumor penetration (Fig. 7A, B). It is worth noting that PGN exhibited the highest fluorescence accumulation in the tumor site, which was 1.46- and 3.43-fold higher than that of PRN and PN, respectively (Fig. 7A, B), highlighting the advantage of the autophagy-responsive ability of GR9.

To further verify the enhanced deep tumor infiltration of PGN, CD31 staining was used to label the tumor blood, and the distribution of NPs was evaluated. As expected, PN showed inferior tumor diffusion activity, while PRN and PGN spread widely around vessel stems, and PGN showed the highest permeability in the three groups (Fig. 7C). Furthermore, the histological images confirmed that the PGN group had the highest fluorescence signal in deep tumor tissues (Fig. 7D). Meanwhile, autophagosomes were labeled by LC3 staining, and we found that the colocalization of PGN and LC3 in tumor tissues was obviously higher than that in the PRN and PN groups (Additional file 1: Fig. S6). These findings indicate that GR9 endows PGNs with deep tumor delivery and autophagy targeting capability in vivo.

### Antitumor efficacy of d-PGN in B16F10 tumor-bearing mice

**Fig. 8:**
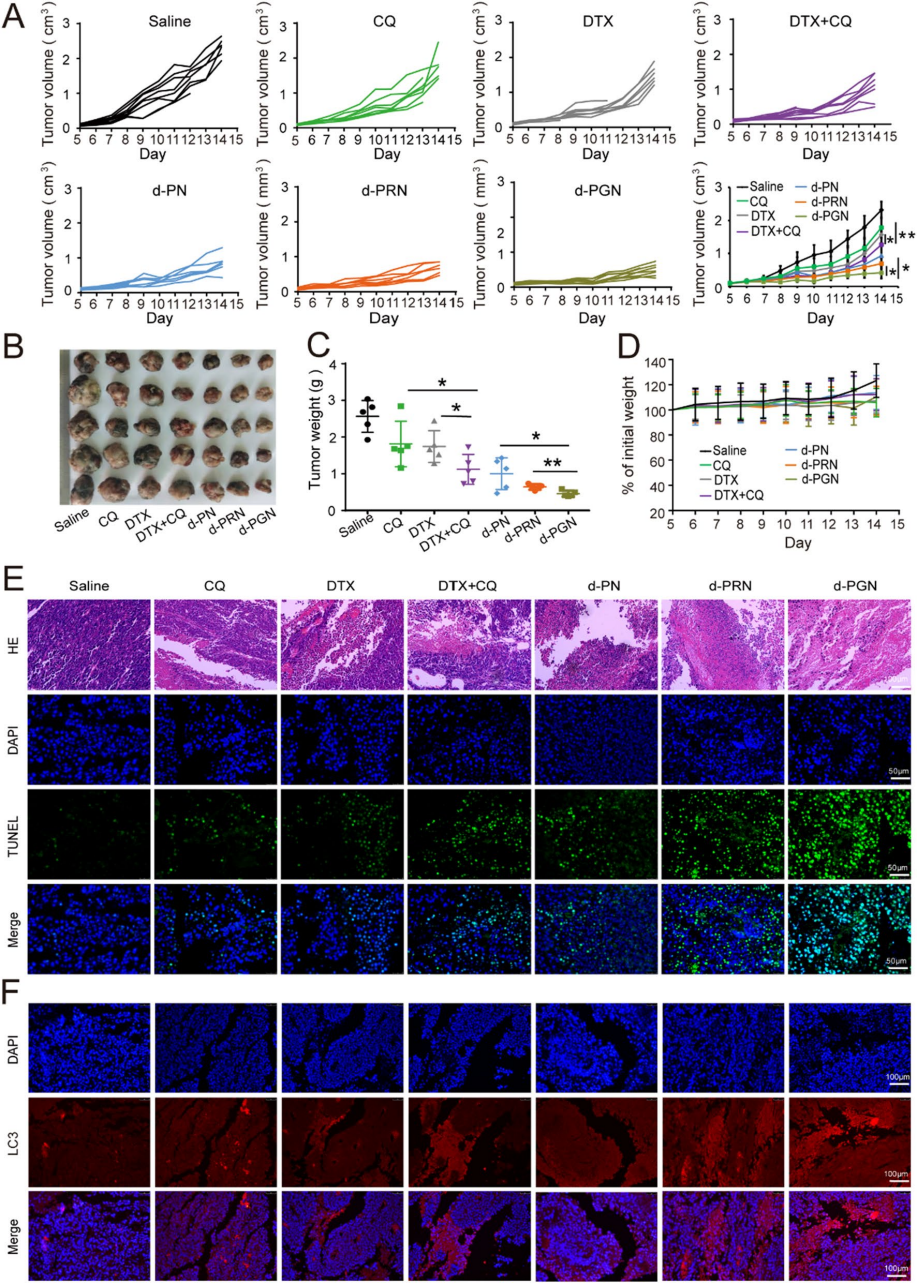
In vivo therapeutic efficacy of PGN in B16F10 tumor-bearing C57 mice. **A** Time-dependent tumor growth curves of mice after treatment with saline, free CQ, DTX, CQ + DTX, d-PN, d-PRN and d-PGN. **B**, **C** Tumors were harvested, and the tumor weight was monitored. **D** The body weight of mice during the treatments. **E** H&E, TUNEL staining of obtained tumor tissues, H&E scale bars: 100 μm, TUNEL scale bars: 50 μm. **F** Analysis of LC3 intensity based on immunofluorescence staining; scale bars: 100 μm. Data are expressed as the mean ± s.d. (n = 7).

The ability of d-PGN to inhibit the growth of the primary tumor was evaluated in B16F10 tumor-bearing C57 mice. The mice were intravenously injected with saline, CQ, DTX, CQ/DTX, d-PN, d-PRN or d-PGN, and the tumor volume and body weight were monitored every day. At the end of the experiment, the B16F10 tumor-bearing mice were sacrificed to harvest the tumors and organs. The results of tumor volume showed that the tumors of the saline group grew rapidly, whereas the growth of the tumors was slightly suppressed by free drug and strongly suppressed by drug loading nanoformulations (Fig. 8A). The tumor picture and tumor weight presented a similar tendency: the nanoformulations presented a higher therapeutic effect than the free drug-treated groups, especially the d-PGN group, which showed the highest tumor inhibition efficacy (Fig. 8B, C). However, no significant changes in body weight were observed during our treatment (Fig. 8D). Hematoxylin-eosin (H&E) staining confirmed that there was no evident histological damage in the tissues of the heart, liver, spleen, lung, and kidneys (Additional file 1: Fig. S7). Moreover, the typical biochemical indicators of the heart, including creatine kinase (CK) and lactate dehydrogenase (LDH); the liver, including aspartate aminotransferase (AST) and alanine aminotransferase (ALT); and the kidney, including creatine (CREA) and urea (UREA), were also examined. The results showed that excluding slight potential damage to the liver and heart by DTX + CQ, all other free drugs and nanoformulations were relatively safe. Notably, in the d-PGN treated group, we did not observe any changes in any biochemical indicators (Additional file 1: Fig. S8). The above results indicate that d-PGN could precisely and effectively deliver drugs into deep tumors, exhibiting excellent antitumor efficacy and biocompatibility in vivo.

Furthermore, the morphological changes in tumor slices were also evaluated by hematoxylin-eosin (H&E) staining. The slices of all free drug- and nanoformulation-treated groups exhibited an obvious reduction in compact nucleated cells and consisted of sparse areas of apoptotic cells, especially in the d-PGN treated tumor slices (Fig. 8E). TUNEL analysis further demonstrated that d-PGN could induce remarkable tumor cell apoptosis, with signs of considerable green fluorescence in cells (Fig. 8E). Additionally, d-PGN treatment dramatically increased the red fluorescence of LC3 immunoreactivity in tumor tissues (Fig. 8F). Taken together, consistent with in vitro experiments, d-PGN released CQ could inhibit autophagosome fusion with lysosomes, resulting in autophagosome accumulation, enhancing the autophagy-responsive sensitivity of GR9, which further improves d-PGN retention in the deep tumor. Additionally, integrating the benefits of enhanced-deep tumor drug delivery and DTX/CQ combination therapy could effectively prevent tumor growth.

## Conclusions

In this study, an autophagy-responsive multifunctional nanoparticle was designed to target deep tumor cells. In vitro experiments showed that the constructed PGN could effectively achieve deep tumor penetration by GR9-mediated high cellular uptake and autophagy-responsive capability. In vivo experiments showed that d-PGN exhibits effective tumor tissue drug delivery capability and excellent antitumor efficacy. Collectively, autophagy-responsive multifunctional nanoparticles provide a novel potential strategy for solid tumor treatment.

## Supplementary Information

**Additional file 1. Supplementary Figures: Figure S1. Analysis of** ^1^H-NMR (A) ^1^H-NMR spectra of DSPE-PEG2000-NHS and (B) DSPE-PEG2000-GR9; ^1^H-NMR spectra of (C) DSPE-PEG2000-NH2 and(D) DSPE-PEG2000-DMA. **FigureS2.** Screening of ratios (DSPE-PEG2000-GR9:DSPE-PEG2000-DMA) to form the desired PGN based on the size and zeta potential of nanoparticles. **Figure S3.** Digital photographs of NPs dispersed in DI water. **Table S1.** Characterizations of drug-loaded NP. **FigureS4.** Hemocompatibility assay. (A) Hemolytic toxicity profile of red blood cells (RBCs) in the presence of d-PN, d-PRN and d-PGN nanoparticles at various concentrations. (B) The hemolysis ratio of each group. **Figure S5.** Confocal microscopy analysis of microtubule aggregation in B16F10 cells after various treatments. Microtubule was labeled by anti-β-tubulin antibody, while nuclei were stained with DAPI. **Figure S6.** The co-localization of Did-labeled nanoparticles and LC3, scale bars: 100 μm. **Figure S7.** H&E stained images of dissected major organs including heart, liver, spleen, lungs and kidneys from different groups for in vivo biosafety evaluation after 14 d treatment, scale bars: 100 μm. **Figure S8.** The typical heart, liver and kidney biochemical indicators of B16F10 tumor-bearing mice after treatment with free drugs or d-NPs. (A) alanine aminotransferase (ALT), (B) aspartate aminotransferase(AST), (C) creatinine (CREA), (D) urea (UREA), (E) creatine kinase (CK), (F)lactate dehydrogenase (LDH). (*P <0.05, **P < 0.01 vs. PBS control).

## Data Availability

All data generated or analyzed during this study are included in this published article and its Additional files.
