# Supplementary material for: Autophagy responsive intra-intercellular delivery nanoparticles for effective deep solid tumor penetration
Source: J Nanobiotechnology. 2022 Jun 25;20:300. doi: 10.1186/s12951-022-01514-6 (PMC9233833; doi:10.1186/s12951-022-01514-6)
Supplement: Supplementary file 1 — Additional file 1. Supplementary Figures: Figure S1. Analysis of 1H-NMR (A) 1H-NMR spectra of DSPE-PEG2000-NHS and (B) DSPE-PEG2000-GR9; 1H-NMR spectra of (C) DSPE-PEG2000-NH2 and(D) DSPE-PEG2000-DMA. FigureS2. Screening of ratios (DSPE-PEG2000-GR9:DSPE-PEG2000-DMA) to form the desired PGN based on the size and zeta potential of nanoparticles. Figure S3. Digital photographs of NPs dispersed in DI water. Table S1. Characterizations of drug-loaded NP. FigureS4. Hemocompatibility assay. (A) Hemolytic toxicity profile of red blood cells (RBCs) in the presence of d-PN, d-PRN and d-PGN nanoparticles at various concentrations. (B) The hemolysis ratio of each group. Figure S5. Confocal microscopy analysis of microtubule aggregation in B16F10 cells after various treatments. Microtubule was labeled by anti-β-tubulin antibody, while nuclei were stained with DAPI. Figure S6. The co-localization of Did-labeled nanoparticles and LC3, scale bars: 100 μm. Figure S7. H&E stained images of dissected major organs including heart, liver, spleen, lungs and kidneys from different groups for in vivo biosafety evaluation after 14 d treatment, scale bars: 100 μm. Figure S8. The typical heart, liver and kidney biochemical indicators of B16F10 tumor-bearing mice after treatment with free drugs or d-NPs. (A) alanine aminotransferase (ALT), (B) aspartate aminotransferase(AST), (C) creatinine (CREA), (D) urea (UREA), (E) creatine kinase (CK), (F)lactate dehydrogenase (LDH). (*P <0.05, **P < 0.01 vs. PBS control). [file 12951_2022_1514_MOESM1_ESM.docx]

**Additional file 1**

**
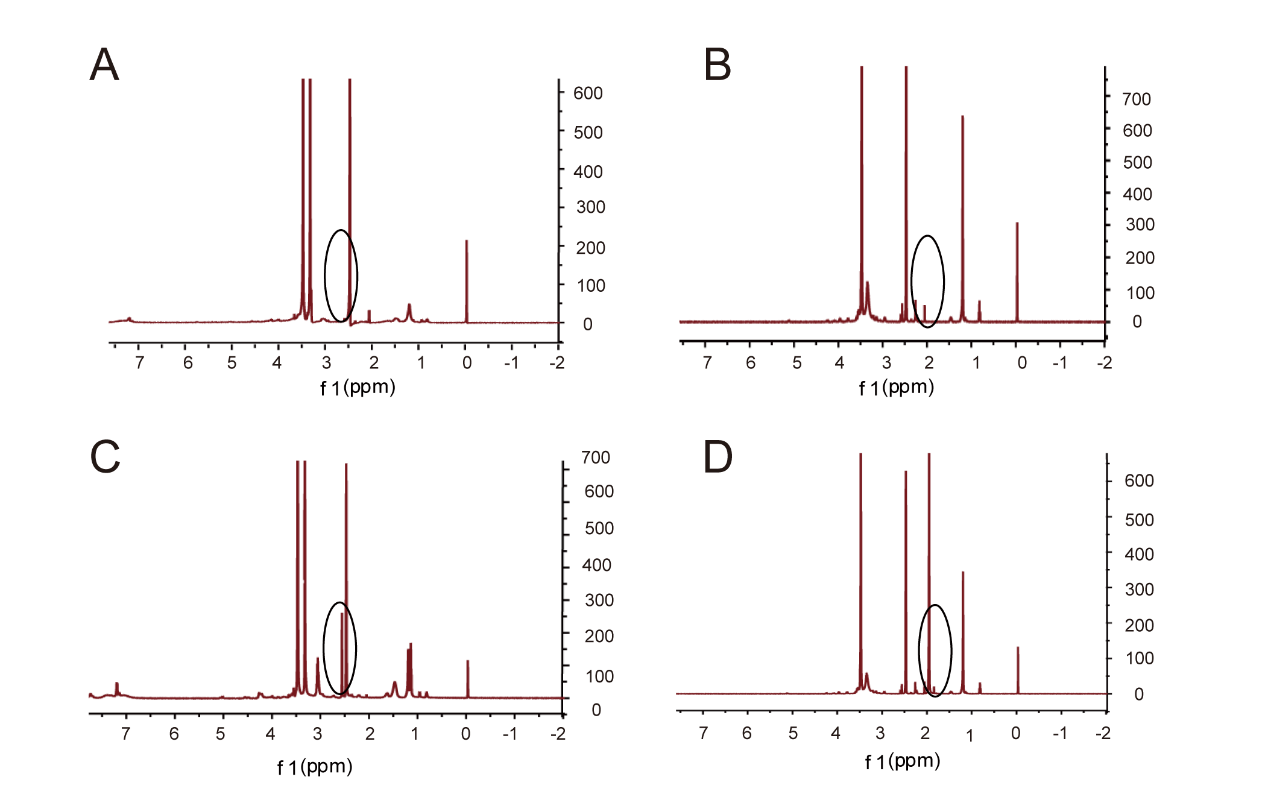
**

**Figure S1.** (A) ^1^H-NMR spectra of DSPE-PEG2000-NHS and (B) DSPE-PEG2000-GR9; ^1^H-NMR spectra of (C) DSPE-PEG2000-NH2 and (D) DSPE-PEG2000-DMA.

**
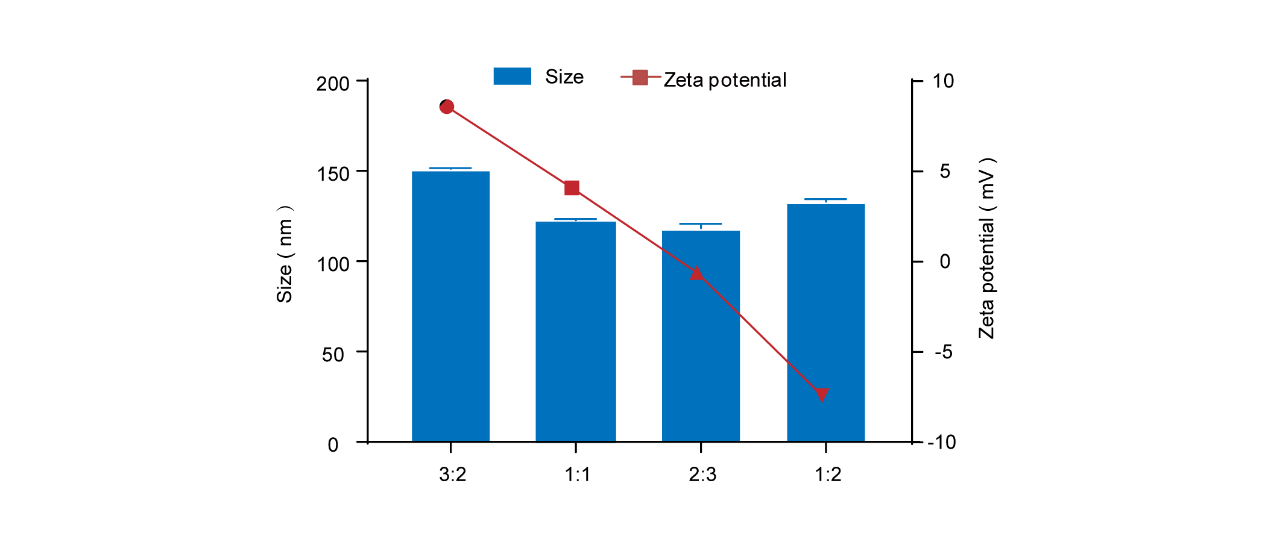
**

**Figure S2.** Screening of ratio (DSPE-PEG2000-GR9: DSPE-PEG2000-DMA) to form desired PGN based on the size and zeta potential of nanoparticles.


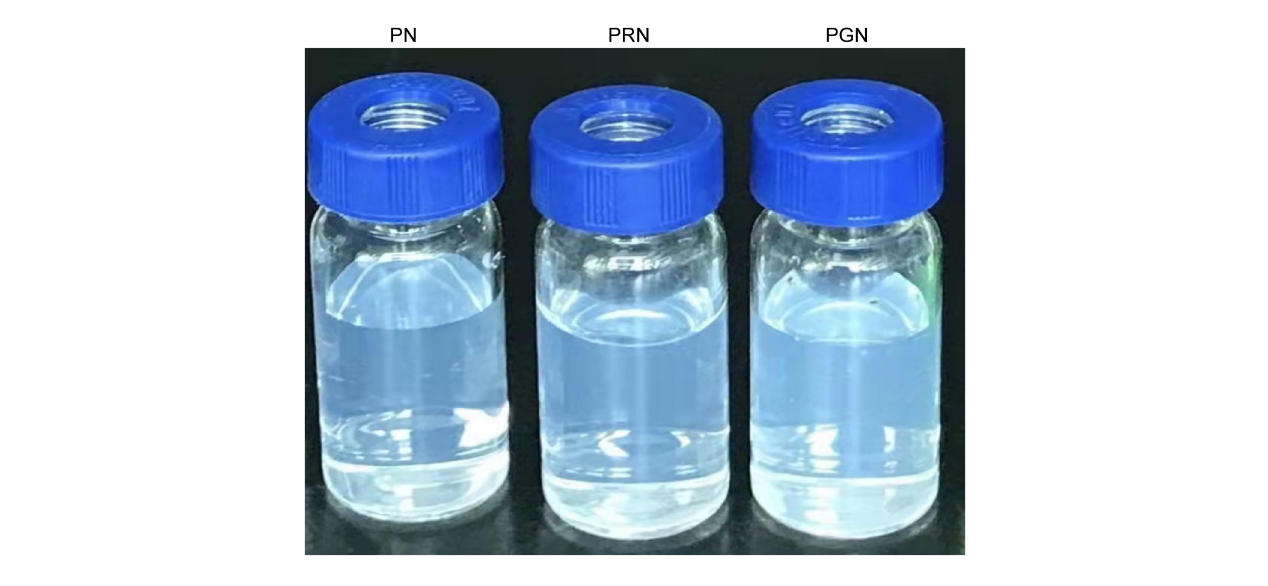


**Figure S3.** Digital photographs of NPs dispersed in DI water.

| Group | Size | PDI | ζ potential | |
| --- | --- | --- | --- | --- |
|  |  |  | pH7.4 | pH6.5 |
| d-PN | 113.0±2.63 | 0.161±0.007 | -11.53±0.21 | -11.3±1.25 |
| d-PRN | 128.8±8.13 | 0.331±0.041 | -0.22±0.35 | 17.73±0.4 |
| d-PGN | 126.5±4.09 | 0.253±0.023 | -2.56±3.06 | 18.97±0.66 |

**Table S1.** Characterizations of drug loaded NP


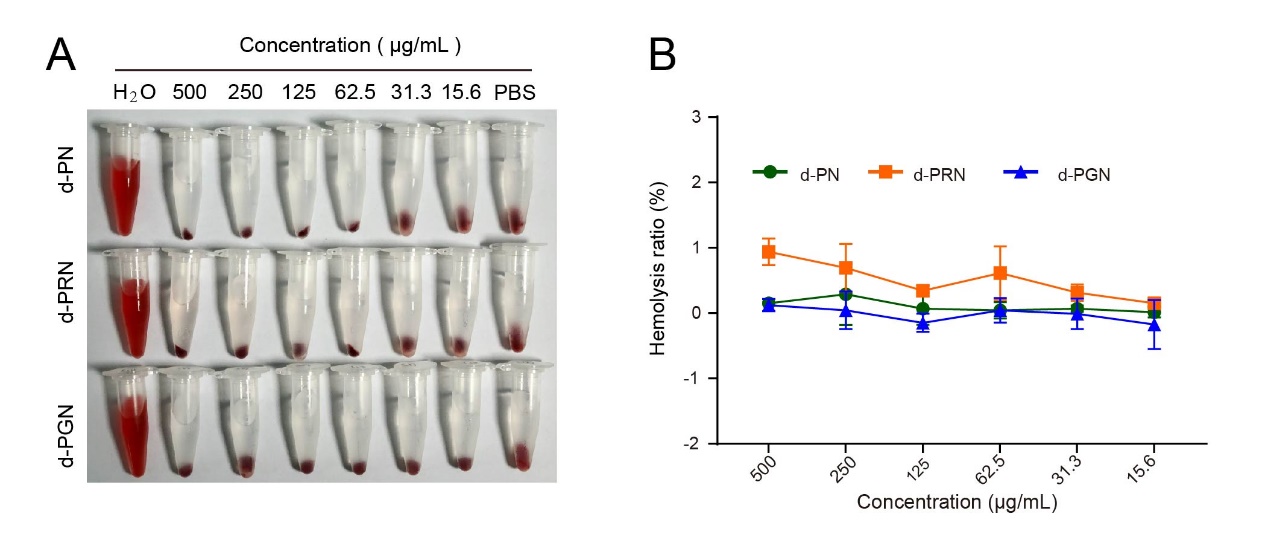


**Figure S4.** Hemocompatibility assay. (A) Hemolytic toxicity profile of red blood cells (RBCs) in the presence of d-PN, d-PRN and d-PGN nanoparticles at various concentrations. (B) The hemolysis ratio of each group.


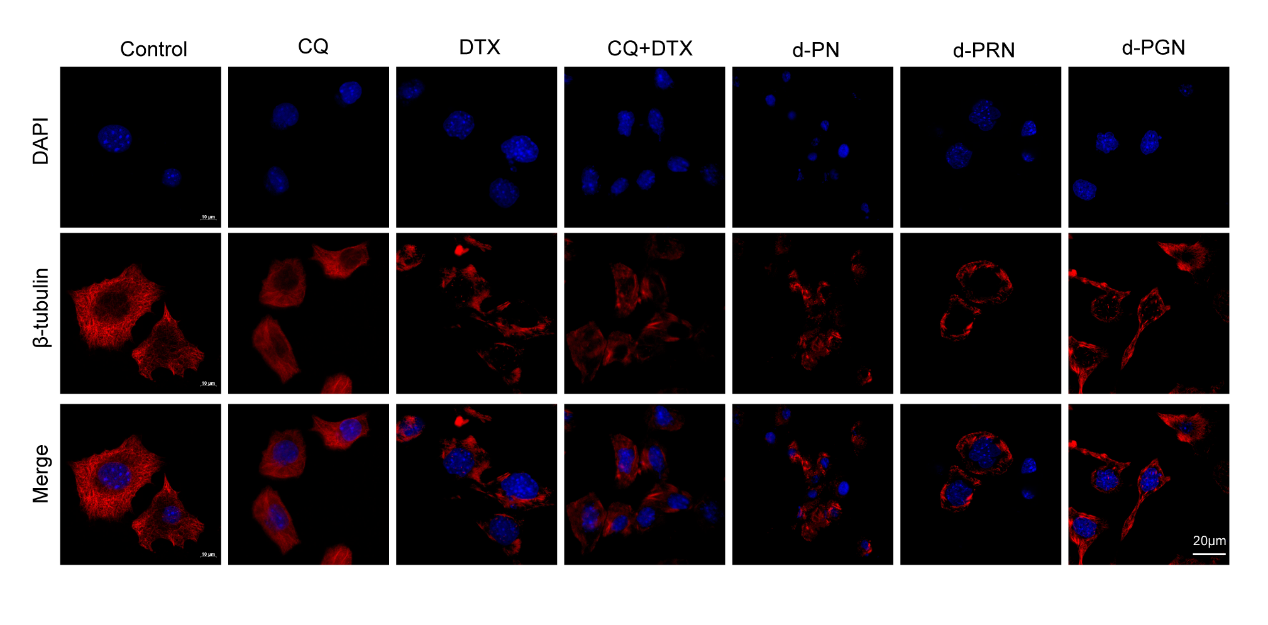


**Figure S5**. Confocal microscopy analysis of microtubule aggregation in B16F10 cells after various treatments. Microtubule was labeled by anti-β-tubulin antibody, while nuclei were stained with DAPI.


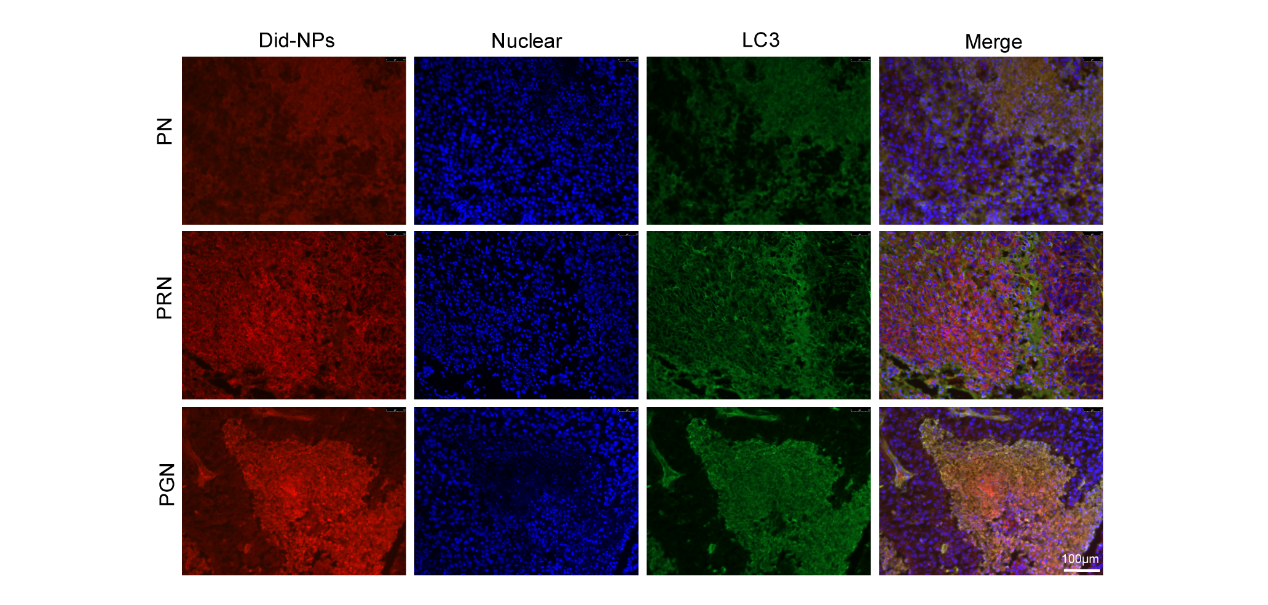


**Figure S6.** The co-localization of Did-labeled nanoparticles and LC3, scale bars: 100 μm.


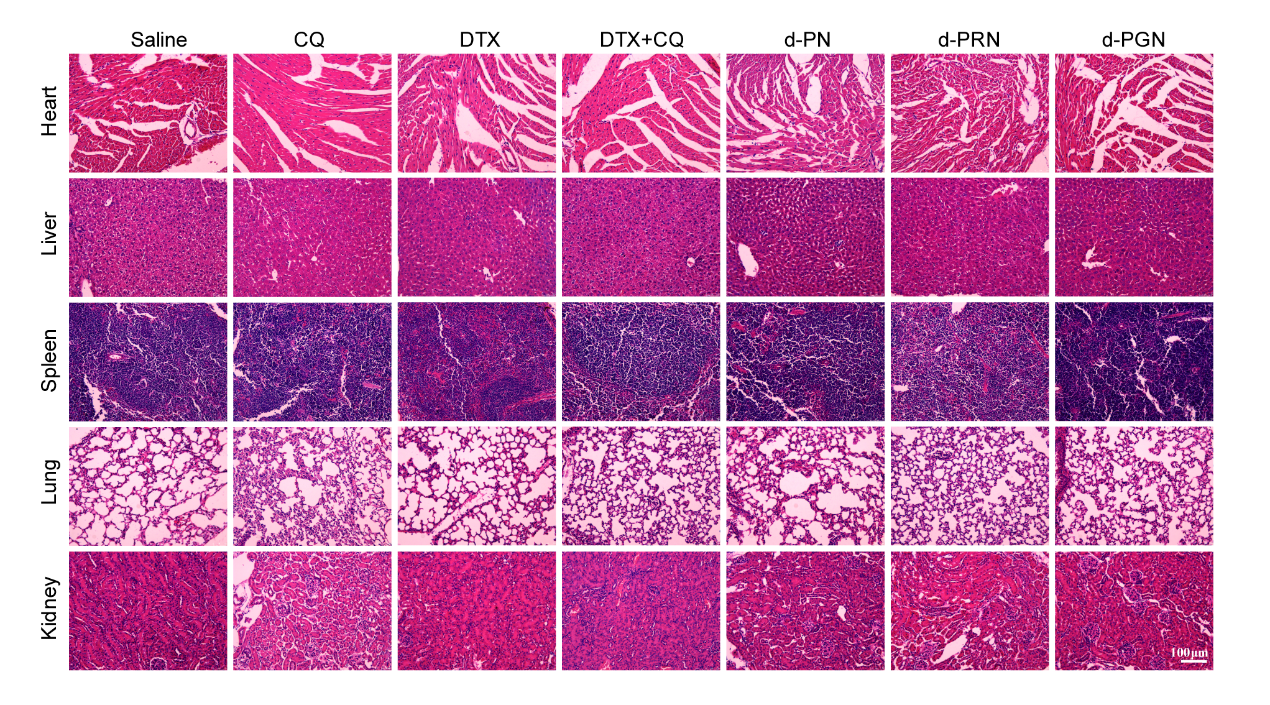


**Figure S7.** H&E stained images of dissected major organs including heart, liver, spleen, lungs and kidneys from different groups for *in vivo* biosafety evaluation after 14 d treatment, scale bars: 100 μm.


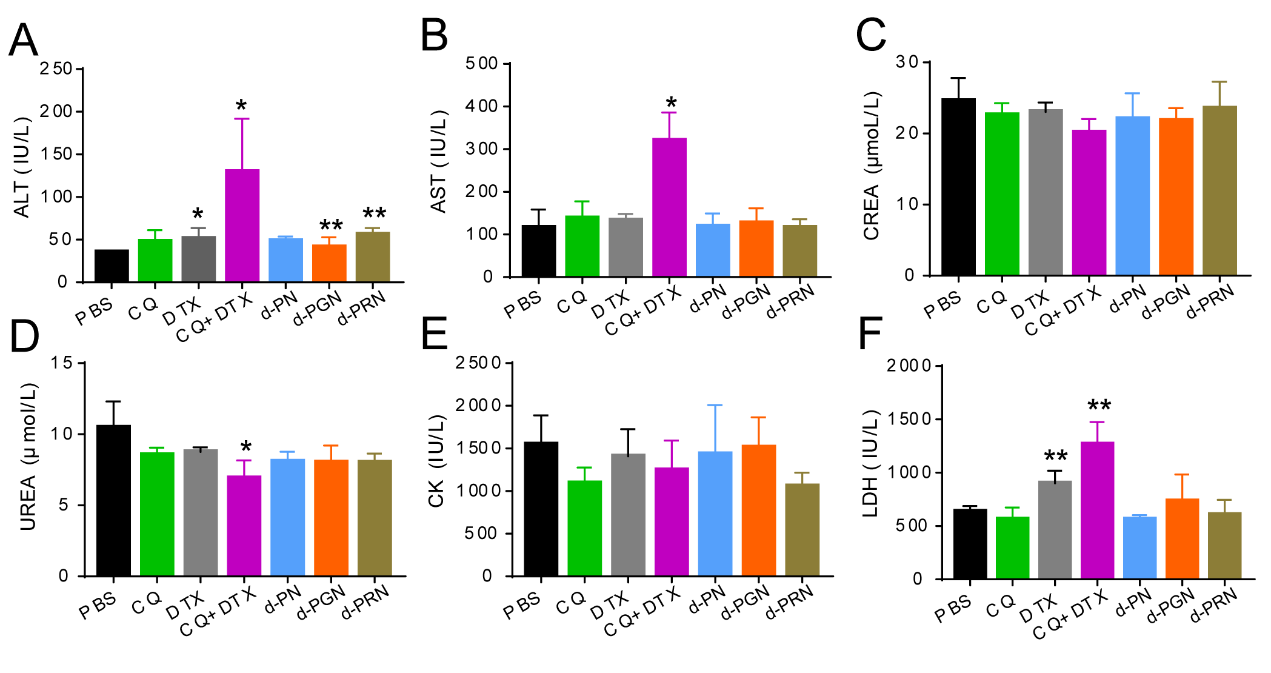


**Figure S8.** The typical heart, liver and kidney biochemical indicators of B16F10 tumor-bearing mice after treatment with free drugs or d-NPs. (A) alanine aminotransferase (ALT), (B) aspartate aminotransferase (AST), (C) creatinine (CREA), (D) urea (UREA), (E) creative kinase (CK), (F) lactate dehydrogenase (LDH). (**P < 0.05*, ***P < 0.01* vs PBS control)
